# Anatomical variations of the anterior communicating artery complex: gender relationship

**DOI:** 10.1007/s00276-014-1313-7

**Published:** 2014-05-22

**Authors:** Roger M. Krzyżewski, Krzysztof A. Tomaszewski, Michał Kochana, Małgorzata Kopeć, Wiesława Klimek-Piotrowska, Jerzy A. Walocha

**Affiliations:** Department of Anatomy, Jagiellonian University Medical College, 12 Kopernika Street, 31-034 Krakow, Poland

**Keywords:** Anterior communicating artery complex, Anterior communicating artery, Anterior cerebral circulations, Anterior cerebral artery, Anatomical variations

## Abstract

**Purpose:**

The anatomy of the anterior communicating artery complex plays a critical role in surgical treatment of anterior cerebral circulation aneurysms. A thorough description of vascular variations of the anterior communicating artery complex seems to be lacking. The aim of this study was to describe the anatomical variations of the anterior communicating artery complex.

**Methods:**

The study group consisted of 411 subjects (52.31 % women), without any intracranial pathologies, that had undergone head computed tomography angiography. We used maximum intensity projections, volume rendering and multi planar reconstructions to study and classify the anatomical variations of the anterior communicating and anterior cerebral arteries.

**Results:**

Male subjects had a significantly higher prevalence of the typical anterior communicating artery complex (59.69 vs. 46.05 %; *p* < 0.01). The aplastic anterior communicating artery (23.26 vs. 15.88 %; *p* = 0.04) and triple A2 segment of the anterior cerebral artery (1.86 vs. 0.00 %; *p* = 0.05) were more common in women than in men.

**Conclusion:**

Female subjects have a higher incidence of variations in the anterior communicating artery complex. There is a higher incidence of anterior communicating artery aplasia among women.

## Introduction

The anterior communicating artery complex consists of two anterior cerebral arteries (ACA), the anterior communicating artery (ACoA) and the recurrent arteries of Heubner [15, 16, 32]. ACA can be divided into the three following segments: A1 originating from the internal carotid artery, A2 extending from ACoA and A3 also known as the pericallosal artery [11, 21]. The ACoA complex has a strong clinical relevance due to the fact that it is the most common site of intracranial aneurysm location [13]. Despite its considerable significance, little is known about the anatomical variations of the ACoA complex.

Many anomalies such as aplasia, hypoplasia, duplication or fenestration of ACA segments and ACoA have been described. Authors used various methods such as digital subtraction angiography, computed tomography angiography or intraoperative observations to study the anterior cerebral circulation. However those studies have a number of limitations. First of all they are focused on patients with intracranial aneurysms [1, 5, 19], and not healthy subjects. Secondly, the authors base their conclusions on a relatively small study group, rarely exceeding 100 patients. Thirdly, their observations are often limited to the anomalies of the A1 segment (most commonly associated with ACoA aneurysms) regardless of ACoA and A2 segment anomalies [1].

Comparatively few studies describe the anatomy of the ACoA complex in subjects without intracranial aneurysms. These are mainly cadaveric studies [26, 27] and thus are hard to extrapolate to living patients. There is still a need to further explore the anatomy of the anterior cerebral circulation. The results of such studies would be useful when planning surgical approaches [25], and would allow to avoid any unexpected anatomical variations during treatment of ACoA aneurysms. Such anatomical problems may include double fenestrations of the A2 segment mimicking an aneurysm neck [18] or mistaking a duplicated A1 segment for an ACoA aneurysm [31].

The aim of this study was to propose a simple classification of ACoA complex configurations using computed tomography angiography (CTA). Furthermore we aimed at determining the possible gender association of ACoA complex anatomical variations.

## Materials and methods

### Study group

The study group was prospectively enrolled over a period of 48 months (December 2009 to December 2013) and consisted of 411 adult subjects (52.31 % female subjects) undergoing head CTA in the Department of Radiology, Jagiellonian University Medical College.

**Fig. 1:**
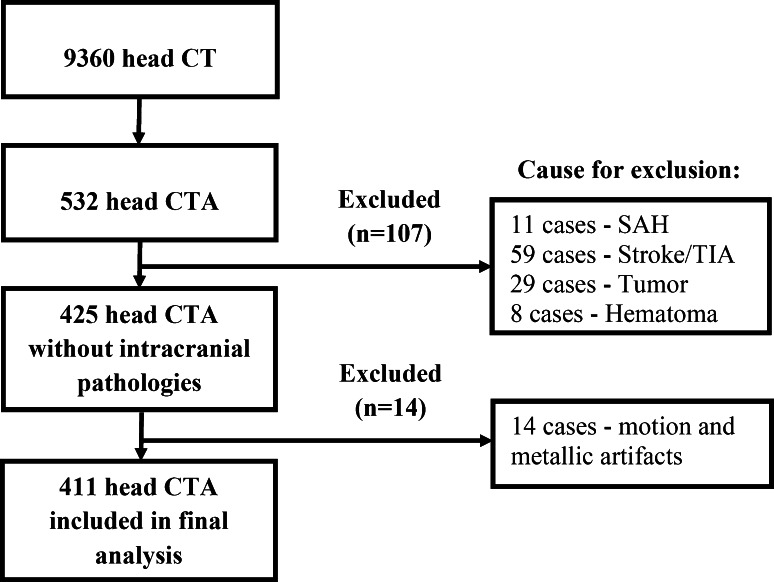
Flowchart depicting the study protocol. *CT* Computed Tomography, *CTA* Computed Tomography Angiography, *SAH* Subarachnoid Hemorrhage, *TIA* Transient Ischemic Attack

The study population was recruited from patients undergoing radiological examination due to a mild head trauma (without an intracranial hematoma) or due to viscerocranial pathologies (i.e. chronic rhinosinusitis). Patient inclusion criteria were: no signs of vascular (aneurysm, cavernous angioma, arteriovenous malformation, etc.) or non-vascular (neoplasm, hematoma, etc.) intracranial pathologies, no history of stroke or intracranial surgical interventions. Detailed information about the exclusion of patients from the study group can be found in Fig. 1. Patients with the presence of motion or metallic artifacts (e.g. due to the presence of a prosthetic metallic filling) were excluded (Fig. 1). Patients were analyzed as an entire group, then divided into two groups according to gender.

Patient informed consent was obtained prior to inclusion into the study. The study protocol has been approved by the Jagiellonian University Bioethics Committee (registry number KBET/299/B/2012).

### Imaging and analysis

Images were acquired using a multi-row computed tomography (Somatom Sensation 16; Siemens AG, Germany) with the following study parameters: exposure, 120 kV, 74 mA, 120 mAs; rotation time, 0.75; slice thickness, 3 mm; pitch, 1.5. Patients were injected intravenously with an iodine contrast medium (Ultravist, Bayer, Germany) to achieve angiographic images. Collected data were transferred to a workstation equipped with IMPAX 6.4 Solution Software (Agfa HealthCare, Belgium). Maximum intensity (MIP), multi planar reconstruction (MPR), and volume rendering (VR) reconstructions were examined in three planes—coronal, sagittal and transverse.

We have carefully examined each part of the ACoA complex and measured the internal diameter of each artery. Arterial segments that were less than 1 mm were classified as hypoplastic [14]. All images were carefully studied by two independent neuroradiologists, with 15 and 20 years of experience. If a difference of opinion on particular patients occurred the patients were examined together by both researchers until consensus was achieved.

### ACoA complex classification

**Fig. 2:**
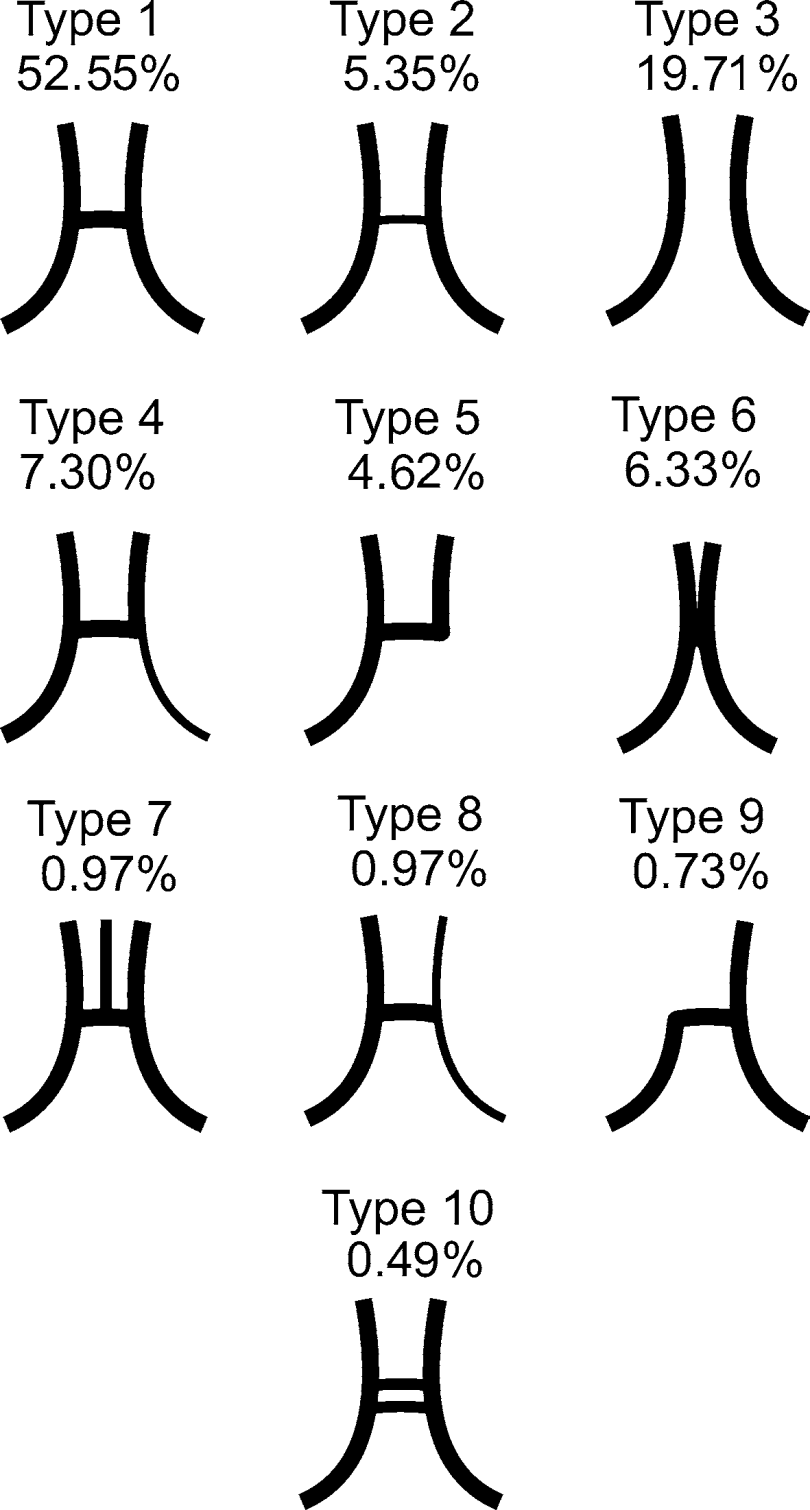
Schematic drawing of the unclassified types of the ACoA complex. *Type 1* typical configuration; *Type 2* hypoplastic ACoA; *Type 3* aplastic ACoA; *Type 4* unilateral *A*1 ACA segment hypoplasia; *Type 5* unilateral *A*1 ACA segment aplasia; *Type 6* common trunk of ACA with absence of ACoA; *Type 7* presence of a third *A*2 ACA segment (median artery of corpus callosum); *Type 8* unilateral hypoplasia of *A*1 ACA segment and *A*2 ACA segment (bihemispheric ACA); *Type 9* unilateral *A*2 ACA segment aplasia (azygos ACA);* Type 10* duplicated ACoA

We classified the ACoA complex into 10 types: Type 1, typical configuration; Type 2, hypoplastic ACoA; Type 3, aplastic ACoA; Type 4, unilateral A1 ACA segment hypoplasia; Type 5, unilateral A1 ACA segment aplasia; Type 6, common trunk of ACA with absence of ACoA; Type 7, presence of a third A2 ACA segment (median artery of corpus callosum); Type 8, unilateral hypoplasia of A1 ACA segment and A2 ACA segment (bihemispheric ACA); Type 9, unilateral A2 ACA segment aplasia (azygos ACA); Type 10, duplicated ACoA. Described types can be seen in Fig. 2. An anatomical variant was assigned to one of the types, if it has been observed in two or more subjects. If a vascular variation was seen in only one subject, it was assigned to the “unclassified” group.

### Statistical analysis

Statistical analysis was conducted using Statistica 10.0 PL by Statsoft. Elements of descriptive statistics were used (mean, standard deviation, percentage distribution). We used *χ* ^2^ Pearson’s test to compare proportions, Student’s *t* test and Mann–Whitney *U* test to compare continuous variables as appropriate. *p* values of less than 0.05 were considered to indicate statistical significance.

## Results

The study group consisted of 411 adult subjects (52.31 % female subjects). The mean age of the group 47.62 ± 18.20 years.

**Fig. 3:**
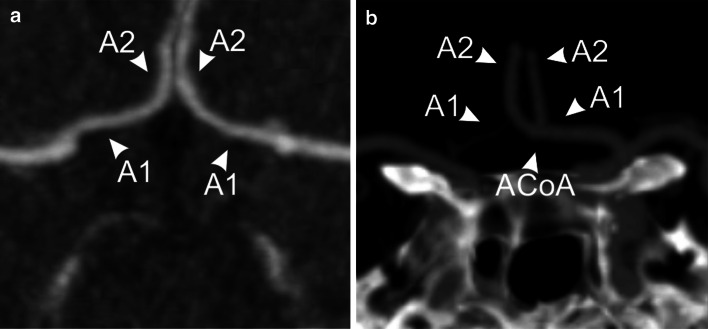
Images showing: a maximum intensity projection image aplastic ACoA (type 3); b multi planar reconstruction image unilateral *A*1 ACA segment hypoplasia (type 4). *A*1 A1 segment of the anterior cerebral artery; *A*2 A2 segment of the anterior cerebral artery; *ACoA* anterior communicating artery

The most common vascular variation was the aplastic ACoA (19.57 % of subjects) and it was more frequent in female subjects (23.15 % vs. 15.66 %; *p* = 0.04) (Fig. 3a). The most frequent variation of the ACA was the A1 segment unilateral hypoplasia (8.94 % of all subjects) (Fig. 3b), but it did not show any differences in gender-related distribution.

**Fig. 4:**
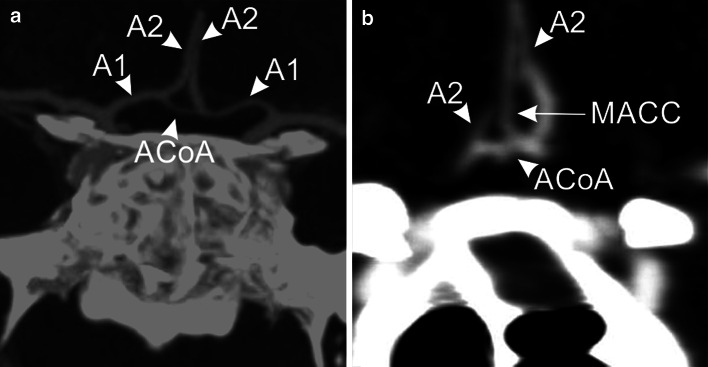
CTA images in transverse plane showing: a multi planar reconstruction image typical variant of the ACoA complex (type 1); b maximum intensity projection image presence of a third *A*2 segment of the anterior cerebral artery (*MACC* median artery of corpus callosum) (type 7). *A*1 A1 segment of the anterior cerebral artery; *A*2 A2 segment of the anterior cerebral artery; *ACoA* anterior communicating artery

According to the classification presented in this manuscript, type 1 occurred more often in men (59.69 % vs. 46.05 %; *p* < 0.01) (Fig. 4a). Type 3 was more prevalent among female subjects (23.26 % vs. 15.82 %; *p* = 0.04). Types 7 and 8 occurred only in female subjects (Fig. 4b), although we observed only borderline statistical significance (*p* = 0.05) (Table 1) (Fig. 2).

**Table 1:** Prevalence of classified variants of the anterior communicating artery complex

Type	Total (*n* = 411)	Female (*n* = 215)	Male (*n* = 196)	*p* value
1 (%)	216 (52.55)	99 (46.05)	117 (59.69)	<0.01
2 (%)	22 (5.35)	15 (6.98)	7 (3.57)	0.12
3 (%)	81 (19.71)	50 (23.26)	31 (15.88)	0.04
4 (%)	30 (7.30)	15 (6.98)	15 (7.65)	0.81
5 (%)	19 (4.62)	12 (5.58)	7 (3.57)	0.33
6 (%)	26 (6.33)	14 (6.51)	12 (6.12)	0.86
7 (%)	4 (0.97)	4 (1.86)	0 (0.00)	0.05
8 (%)	4 (0.97)	4 (1.86)	0 (0.00)	0.05
9 (%)	3 (0.73)	2 (0.93)	1 (0.51)	0.61
10 (%)	2 (0.49)	0 (0.00)	2 (1.02)	0.13
Unclassified	4 (0.97)	0 (0.00)	4 (2.04)	0.03

*p* value of comparison between female and male

Four cases of ACoA complex configuration were not assigned to any of the aforementioned types. These were: unilateral A1 ACA segment fenestration, A2 ACA segment fenestration, hypoplasia of the A1 segment and the contrlateral A2 segment, unilateral ACA (aplasia of ACoA and contrlateral aplasia of both ACA segments). Unclassified segments were exclusive to male subjects (2.04 vs. 0.00 %; *p* = 0.03) (Table 1).

## Discussion

In this prospective study we have described anatomical variations of the ACoA complex using CTA. Male subjects more frequently had a typical variant of the ACoA complex. Female subjects more often had an aplastic ACoA. Other variations had borderline statistical significance.

Our study found only 0.48 % (2 cases) of arterial fenestration which is strikingly low in comparison to other studies. Autopsy studies described the frequency of fenestrations in the anterior circulation in up to 64.4 % [25]. Investigations that used three-dimensional digital subtraction angiography (DSA) found a greater incidence of fenestrations in the anterior cerebral circulation, which was 27 % for aneurysm patients and 22 % for patients without aneurysm [30]. On the other hand, Sanders et al. studying 5,190 cerebral angiograms found only 3 fenestrations in the ACoA complex [23]. A study by Bożek et al. using CTA described the incidence of ACoA complex fenestrations to be 1.75 % [2]. These findings emphasis the fact that two-dimensional imaging is not a suitable tool for detecting intracranial arterial fenestrations.

The most common anomaly of the anterior cerebral circulation was an aplastic ACoA which was more prevalent in females. Cadaveric studies show a vast range of ACoA hypoplasia frequency from 9.15 to 30 % [6, 12, 22]. In addition, aplastic ACoA is a rare autopsy finding, found only in 1.8 % of studied subjects [9]. This phenomenon can be explained by the fact that hypoplastic arteries may not be hemodynamically efficient, therefore are not visible in angiographic studies and thus are considered to be aplastic. On the other hand, autopsy findings always visualize the artery trunk, even when contrast flow would not be possible. Li et al. using CTA found aplastic ACoA’s in 9.38 % subjects [14]. ACoA 3D imaging provides the excellent quality data, but unfortunately is rarely available in everyday clinical practice [33].

In our material we found four cases of a triple A2 segment, which was exclusive to female subjects. Most authors identify the triple A2 segment as a persistent median artery of corpus callosum, a remnant of embryological cerebral circulation [17]. MRA studies show that the frequency of a triple A2 segment ranges between 0.4 and 3.03 % [20, 28, 29]. Usually a triple A2 is an incidental finding. Sun et al. reported a very interesting case of a triple A2 segment associated with the presence of an aneurysm [24]. We have not found such a variant in our study.

The A1 and A2 segments of ACA are clinically most significant. Many authors associated the presence of an A1 segment aplasia or hypoplasia with the presence of an ACoA aneurysm. Castro et al. [4] found an association between an asymmetric A1 artery blood inflow, ACoA aneurysm formation and rupture. Aplastic or hypoplastic A1 segment of ACA is a common finding in patients with ACoA aneurysms and can be considered as risk factor for aneurysm formation [5, 10]. The frequency of A1 segment hypoplasia in patients without aneurysms ranges between 4.76 and 8.41 % (this study 7.25 %). Aplasia of the A1 segment is relatively less frequent 1.86–8.0 % (this study 4.59 %) [5, 10, 28]. There are few clinically important variations of the ACA that we have not found in our study, e.g. an inter-optic or an infra-optic course of ACA [3, 8].

This study is not free of certain limitations. First of all, in some cases, a 16-row CTA may not be sufficient enough to detect very small abnormalities. Hence the relatively low frequency of detected arterial fenestrations. However, we have to bear in mind that the quality of images obtained using a Somatom Sensation 16 is still considered diagnostically efficient, and provides clinically relevant information. Though we confirm that the use of a 64-detector CT might bring to light additional information that we were not able to obtain in the present study [7]. Cadaveric studies are more detailed in nature, although they may often describe hemodynamically closed arteries. Secondly, we described our findings on a relatively small patient group, not exceeding 1,000 subjects. On the other hand, according to our best knowledge, this group is the largest among studies assessing the ACoA complex using CTA. Thirdly, we did not analyze CTA source images that allow to visualize very small arteries. Hence some of the hypoplastic arteries might have been misdiagnosed as aplastic arteries.

Our findings may be particularly interesting for neurosurgeons operating in the vicinity of the ACoA complex. Patients with an A1 or A2 segment hypoplasia or aplasia may by more prone to ischemic complications after surgery, i.e. ACoA aneurysm clipping. Triple A2 or fenestration in the A2 segment may be mistaken for an aneurysm during digital imaging [23]. A lack of the ACoA disrupts the circulation in the Circle of Willis, putting patients at risk of ischemic complications. The presented results suggest that neurosurgeons should be more alert whilst operating females, seeing that they have a higher frequency of anatomical abnormalities in the ACoA complex.

Concluding, in this study we have presented the anatomical variations occurring in the ACoA complex. Moreover, we have proposed a simple classification system regarding the mentioned variations.
